# IL-12 superfamily members guiding the function of Rorγt-dependent innate lymphocytes

**DOI:** 10.1186/1479-5876-10-S3-I15

**Published:** 2012-11-28

**Authors:** Burkhard Becher

**Affiliations:** 1 Institute of Experimental Immunology, University of Zurich, Switzerland

## 

Most MHC class II expressing Ag-presenting cells (APCs) have the capacity to produce the cytokines IL-12 and IL-23 [1]. Both these heterodimeric pro-inflammatory cytokines share a common subunit (p40) which is covalently linked to p35 to form IL-12 and to p19 to form IL-23. The biology of these two related cytokines is extremely divers. IL-12 is best known for its capacity to polarize T_H_1 cells and to activate NK and NKT cells. IL-12 is thus primarily involved in the initiation of cellular immune responses agains intracellular pathogens. The biology of IL-23 is much less understood, but it becomes increasingly clear that IL-23 can activate innate lymphocytes including a subclass of γδ T cells [2] and Rorγt-dependent innate lymphocytes (ILCs) [3]. IL-23 is further critical for the development of self-reactive pathogenic αβ helper T cells in various models of autoimmune diseases [4].

In the context of anti-tumor immunity we discovered that IL-23 plays only a minor role in the development of anti-tumor responses. In fact, we found IL-23 to have primarily tumor-supportive properties. In contrast, IL-12 has clearly a potent tumor-suppressive properties. Surprisingly, we identified a Rorγt-dependent IL-12R bearing ILC homing into the microenvironment of skin tumors [5]. These ILCs upon sensing IL-12 are able to mount a potent innate response in the tumor-microenvironment, leading to alterations in tumor microvessels and the formation of a pro-inflammatory myeloid cell response. Taken together, our initial understanding of IL-12 and IL-23 biology was restricted to adaptive T cells. The impact of these cytokines on γδ T cells and ILCs is only now being discovered.
